# Successful Production of Offspring Derived from Phospholipase C Zeta-Deficient Sperm by Additional Artificial Activation

**DOI:** 10.3390/life13040980

**Published:** 2023-04-10

**Authors:** Naoki Hirose, Yasuyuki Kikuchi, Atsuko Kageyama, Hibiki Sugita, Miu Sakurai, Yui Kawata, Jumpei Terakawa, Teruhiko Wakayama, Junya Ito, Naomi Kashiwazaki

**Affiliations:** 1 Faculty of Life and Environmental Science, University of Yamanashi, Yamanashi 400-8510, Japan; 2 Laboratory of Animal Reproduction, Graduate School of Veterinary Medicine, Azabu University, Kanagawa 252-0206, Japan; 3 School of Veterinary Medicine, Azabu University, Kanagawa 252-0206, Japan; 4 Advanced Biotechnology Center, University of Yamanashi, Yamanashi 400-8510, Japan; 5 Center for Human and Animal Symbiosis Science, Azabu University, Kanagawa 252-0206, Japan

**Keywords:** oocyte activation, sperm factor, phospholipase c, calcium, fertilization

## Abstract

During mammalian fertilization, repetitive rises of intracellular calcium called calcium oscillations are required for full activation of oocytes. Therefore, oocytes such as round spermatid injected or somatic cell nuclear transferred require additional artificial activation which mimics the calcium oscillations. It is well recognized that sperm specific phospholipase C (PLCζ) is a strong candidate as the sperm factor which can induce calcium oscillations and, at least in mammals, the genetic mutation of PLCζ in human causes male infertility due to the lack of calcium oscillations in the oocytes. Recent studies showed that the sperm lacking PLCζ (*Plcz1^−/−^*) still could induce rise(s) of intracellular calcium in the oocytes after IVF but not intracytoplasmic sperm injection (ICSI). In the ICSI oocytes, no pronuclear formation or development to the two-cell stage was observed. However, it is still unclear whether additional activation treatment can rescue the low developmental ability of *Plcz1^−/−^*-sperm-derived oocytes after ICSI. In this study, we examined whether oocytes injected with a *Plcz1^−/−^* sperm can develop to term by additional artificial activation. In oocytes injected a *Plcz1^−/−^* sperm and *Plcz1^−/−^* and *eCS* (another candidate of the sperm factor) double knockout sperm (*Plcz1^−/−^eCS^−/−^*), the rates of pronuclear formation were very low (2.0 ± 2.3% and 6.1 ± 3.7%, respectively) compared to control (92.1 ± 2.6%). However, these rates were dramatically improved by additional procedures of PLCζ-mRNA injection or SrCl_2_ treatment (*Plcz1^−/−^* sperm + PLCζ mRNA, *Plcz1^−/−^* sperm + SrCl_2_ and *Plcz1^−/−^eCS^−/−^* sperm + PLCζ mRNA; 64.2 ± 10.8%, 89.2 ± 2.4% and 72.6 ± 5.4%, respectively). Most of the oocytes were developed to the two-cell stage. After embryo transfer, healthy pups were obtained in all these groups (*Plcz1^−/−^* sperm + PLCζ mRNA:10.0 ± 2.8%, *Plcz1^−/−^* sperm + SrCl_2_:4.0 ± 4.3% and *Plcz1^−/−^eCS^−/−^* sperm + PLCζ mRNA: 10.0 ± 5.7%). The rate in *Plcz1^−/−^* sperm + SrCl_2_ group was significantly lower than that in control (26.0 ± 2.4%). Taken together, our present results show that additional activation treatment such as SrCl_2_ and PLCζ mRNA can fully support to develop to term even in oocyte injected *Plcz1^−/−^* sperm. In addition, PLCζ-induced oocyte activation is more suitable for successful development to term compared to that such as phenomenon induced by SrCl_2_. These findings will contribute to improvement for male-dependent human infertility and reproductive technologies in other mammalian species.

## 1. Introduction

During mammalian fertilization, the penetration of sperm into the ooplasm triggers repetitive increases in the intracellular calcium concentration, i.e., ‘calcium oscillations.’ These oscillations induce events such as the resumption of meiosis at the metaphase-II (MII) stage, the exocytosis of cortical granules, and the formation of the pronucleus (PN), which together are called ‘oocyte activation’ [1,2,3]. It has thus been expected that calcium oscillation-dependent oocyte activation is indispensable for development from embryo to term.

Saunders and his colleagues [4] proposed that phospholipase Cζ (PLCζ) might be the sperm-derived oocyte-activating factor that can induce calcium oscillations during fertilization in the mouse. A microinjection of RNA encoding PLCζ [4] or recombinant PLCζ protein [5] induced calcium oscillations. In addition, the calcium oscillation-inducing activity of sperm extracts was lost when the sperm extracts were pretreated with an antibody against PLCζ [4]. The level of PLCζ that is able to produce fertilization-like calcium oscillations in a single oocyte is in the same range as the single-sperm content of PLCζ [4]. To date, PLCζ has been detected in many mammalian and some non-mammalian species, including mouse [4,6], rat [5], human [5,7], cynomolgus monkey [7], cow [8,9], pig [10,11], horse [12] and medaka fish [5]. Taken together, at least in mammals, it has been believed that PLCζ is a strong candidate as the sperm factor which conserved in many mammalian species. The sperm-induced calcium oscillations are generated by a release of calcium from 1,4,5-inositol trisphosphate (IP_3_)-sensitive intracellular calcium stores [13,14]. Since IP_3_ is generated by the PLCζ-mediated hydrolysis of phosphatidyl inositol 4,5-bisphosphate (PI(4,5)P_2_), the activation of PLCζ and the production of IP_3_ are required for the induction of calcium oscillations. Genetic mutations of PLCζ cause male infertility in humans [15,16,17,18]. These findings strongly suggest that PLCζ functions as a sperm factor that induces calcium oscillations, and that PLCζ is thus indispensable for oocytes’ activation and further development, at least in mammals.

Two research groups have reported their production of a *Plcz1* (the gene name of PLCζ)-deficient (*Plcz1^−^*^/*−*^) mouse [19,20]. Surprisingly, both groups reported that the *Plcz1^−^*^/*−*^ male mice were not infertile and that their sperm can fertilize oocytes even with a lack of calcium oscillations. These results led us to hypothesize that PLCζ is dispensable for oocytes’ activation and further embryonic development. It was also reported that oocytes were activated when *Plcz1^−^*^/*−*^ sperm were used for in vitro fertilization, but not when intracytoplasmic sperm injection (ICSI) was applied [19,20], suggesting that oocyte activation via the fusion of the oocyte membrane is possible even in *Plcz1^−^*^/*−*^ sperm.

Many reproductive technologies such as ICSI and round spermatid injection (ROSI) have been developed and used in the study of human reproduction. The successful production of cloned animals derived from somatic cell nuclear transfer (SCNT) was reported in several mammalian species [21,22,23,24]. Since it is known that the injection of a round spermatid cannot induce the activation of oocytes and since sperm are not used for SCNT, it is necessary to devise artificial activation procedures that mimic the rise(s) of intracellular calcium at fertilization in order to achieve oocyte activation. In studies seeking to accomplish the artificial activation of oocytes in mice, strontium chloride (SrCl_2_) is used for technologies such as ROSI [25] and SCNT [26] because it is believed that SrCl_2_ treatment can produce fertilization events such as calcium oscillations in oocytes [27]. However, it has not been known whether SrCl_2_ treatment can induce oocyte activation and subsequent embryonic development in addition to calcium oscillations at fertilization, or whether oocytes injected with *Plcz1^−^*^/*−*^ sperm can develop to term when subjected to artificial activation.

In addition to PLCζ, it is thought that extra-mitochondrial citrate synthase (eCS) is another known candidate as the sperm factor inducing oocyte activation [28]. CS is a core enzyme of the mitochondrial tricarboxylic acid cycle, which directly controls cellular function [28]. Previous reports showed that eCS is produced and abundant in the sperm head and the injection of eCS mRNA induced calcium oscillations in mouse oocytes [28]. eCS deficient (*eCS^−/−^*) male mice were initially fertile but calcium oscillations were delayed in oocytes fused with eCS deficient (*eCS^−/−^*) sperm, despite normal expression of PLCζ [28]. In addition, *eCS^−/−^* males were fertile, but their litter size was remarkably reduced in 6-month-old males [28]. These results suggest eCS also may have a role in calcium oscillations, oocyte activation and male fertility independent of PLCζ. However, the effect of deletion of both *PLCz1* and *eCS* on oocyte activation still remains unclear.

We conducted the present study to determine whether SrCl_2_ treatment or PLCζ mRNA injection after ICSI using *Plcz1^−^*^/*−*^ sperm can restore oocyte activation and the subsequent embryonic development. We also examined whether *Plcz1^−/−^eCS^−/−^* sperm can induce oocyte activation or not when the sperm was used for ICSI.

## 2. Materials and Methods

### 2.1. Materials

All chemicals and reagents were purchased from Sigma-Aldrich (St. Louis, MO, USA) unless otherwise stated.

### 2.2. Animals

The mice used in this study were C57BL/6 female mice (8–10 weeks of age) for the collection of metaphase II (MII) oocytes, and C57BL/6 males (12–24 weeks of age) were used for the sperm collection. The female mice were purchased from SLC Inc. (Hamamatsu, Japan). Mature female ICR mice (12–14 weeks of age) were used as recipients of the embryo transfer. Vasectomized male ICR mice (20–30 weeks of age) were used to induce pseudopregnancies. The surrogate pseudopregnant ICR females that were used as recipients of the embryos were mated with vasectomized ICR males whose sterility had been confirmed. *Plcz1^+^*^/*−*^ [20] and *eCS^+^*^/*−*^ mice [28] were transferred from RIKEN BioResource Research Center (Ibaraki, Japan) and National Center for Child Health and Development (Tokyo, Japan), respectively. *Plcz1^−^*^/*−*^*eCS^−^*^/*−*^ double knock out mice were produced by crossing with *Plcz1^+^*^/*−*^ and *eCS^+^*^/*−*^ mice.

On the day of the experiments or after all experiments were completed, the mice were sacrificed by CO_2_ inhalation or cervical dislocation and used for further experiments. The mice were housed in an environmentally controlled room with a 12-h dark/12-h light cycle at a temperature of 23 ± 2 °C and humidity of 55 ± 5% with free access to a laboratory diet and filtered water.

### 2.3. Histology

Testes were collected from C57BL/6 (WT), *Plcz1^−^*^/*−*^ and *Plcz1^−^*^/*−*^*eCS^−^*^/*−*^ male mice (N ≥ 3 each) after euthanasia and then fixed with 4% paraformaldehyde (PFA) solution in phosphate-buffered saline (PBS) overnight at 4 °C. Fixed testes were dehydrated through an ethanol gradient and embedded in the paraffin using the Cell & Tissue Processor CT-Pro20 (GenoStaff, Tokyo, Japan) and the HistoStar workstation (Thermo Fisher Scientific, Waltham, MA, USA). Paraffin sections measuring 6 µm were prepared on an RX-860 microtome (Yamato Kohki Industrial, Saitama, Japan) and stained with hematoxylin and eosin (H&E) according to the standard procedures for histological evaluation. Spermatogonia, spermatocytes, round spermatids, and elongating/elongated spermatids were evaluated morphologically. Photomicrographs were taken with a BZ-X700 microscope (Keyence, Osaka, Japan). The representative image is shown.

### 2.4. Sperm Cryopreservation and Sperm Motility Analysis

Spermatozoa obtained from the cauda epididymis were counted and then cryopreserved. To thaw sperm, a straw was warmed for 1 min in water bath (37 °C) after keeping for 10 sec in air. After thawing, spermatozoa were incubated in a drop of Human Tubal Fluid (HTF) medium at 37 °C under 5% CO_2_. Forty-five minutes after incubation, spermatozoa were collected from the top of the drop. Sperm motility was analyzed using the SMAS sperm motility analysis system (DITECT Corporation, Tokyo, Japan).

### 2.5. Oocyte Preparation

Superovulation was conducted as previously described [29]. Cumulus oocyte complexes (COCs) at the metaphase-II stage were collected from the oviducts of the mice that were superovulated by an i.p. injection of 5 IU equine chorionic gonadotropin (eCG; Nippon Zenyaku Kogyo, Tokyo, Japan) followed by 5 IU human chorionic gonadotropin (hCG; Asuka Pharmaceutical Co., Tokyo, Japan) 48 h later. Twelve to fourteen hours after hCG injection, the females were sacrificed and their oviductal ampullae were removed. The oviductal ampullae were placed in oil, and COCs were collected from the oviductal ampullae and then transferred to HEPES-buffered CZB (HEPES–CZB) medium [30]. To disperse the cumulus, the COCs were transferred into a 100-μL droplet of HEPES-CZB medium containing 0.1% hyaluronidase (Sigma, St. Louis, MO, USA) for 3 min. The cumulus-free oocytes were washed twice and then moved to a 20-μL droplet of CZB medium [31] for further experiments.

### 2.6. Preparation of Mouse PLCζ mRNA and Its Microinjection

We used pCS2^+^-*mousePlcz1* [10] as the template for mRNA synthesis. mRNA was synthesized as described. The in vitro transcription was carried out using an mMESSAGE MACHINE sp6 kit (Life Technologies, Carlsbad, CA, USA) according to the manufacturer’s instructions. The synthesized RNA was polyadenylated with a poly(A) tailing kit (AM1350, Life Technologies). The RNA with poly(A) tail was precipitated using lithium chloride and dissolved in nuclease-free water. After the concentration was measured with a spectrophotometer (NanoDrop ND-1000, NanoDrop Technologies, Wilmington, DE, USA), 500 ng/μL aliquots were stored at −80 °C until further use.

Before microinjection, PLCζ mRNA was diluted with nuclease-free water to 1 ng/µL. The microinjection (~10 pL/oocyte) of mRNA into oocytes was executed using a piezo-driven micropipette (Prime Tech, Ibaraki, Japan) as previously reported [29]. Briefly, each microinjection was performed in HEPES-CZB medium on an inverted microscope (Olympus, Tokyo, Japan) with a micromanipulator (Narishige, Tokyo, Japan). The zona pellucida and cytosolic membrane were penetrated with a piezo drive. After the mRNA injection, the oocytes were kept in HEPES-CZB medium at room temperature for 10 min before culturing in CZB medium.

### 2.7. Production of Fertilized Embryos by Intracytoplasmic Sperm Injection

ICSI was performed as described [30,32]. The frozen/thawed sperm were retrieved into HEPES-CZB medium and were washed three times with the same medium to remove cryoprotectant. The frozen sperm head derived from B6 (WT), *Plcz1^−^*^/*−*^, and *Plcz1^−^*^/*−*^*eCS^−^*^/*−*^ male mice was separated from the tail by the application of several piezo pulses to the head-midpiece junction (the neck) in 10% Polyvinylpyrrolidone Solution with HAS (FUJIFILM Wako Chemicals, Tokyo, Japan). The separated head was then injected into the oocyte collected from a WT female mouse in HEPES-CZB medium. After 10 min of recovery at room temperature, the oocyte was cultured in CZB medium for preimplantation development. For SrCl_2_ treatment, some of the sperm-injected oocytes were activated using 5 mM SrCl_2_ in Ca^2+^-free CZB medium for 30–60 min.

### 2.8. In Vitro Culture and Embryo Transfer

After the ICSI, mRNA injection, and SrCl_2_ treatment, the morphologically-survived oocytes were incubated in CZB medium at 37 °C in 5% CO_2_. Pronuclear formation was checked 6 h after activation/injection. The embryos were washed three times in KSOM-AA and then transferred into 100 µL of KSOM-AA and were cultured up to blastocysts at 37.5 °C under 5% CO_2_ in air. Cleavage and blastocyst formation of the oocytes were evaluated at 18 h and 114 h post-ICSI, respectively. For the experiments of embryo transfer, embryos at the two-cell stage were transferred to a day-0.5 pseudopregnant mouse that had been mated with a vasectomized male the night before transfer. After, approximately 10–15 embryos were transferred into each oviduct. At day 18.5 of gestation, the offspring were delivered by caesarean section and allowed to mature. Offspring rate, offspring weight and placenta weight were examined.

### 2.9. Immunostaining

Blastocysts were used for the immunofluorescence staining applied to evaluate the quality of the embryos [32]. Following three washes in PBS containing 0.1% polyvinyl alcohol (PBS-PVA), blastocysts were fixed for 60 min in PBS-PVA containing freshly prepared 2% (*w*/*v*) paraformaldehyde and 0.2% (*v*/*v*) Triton X at 24 °C. After fixation, oocytes were washed with PBS-PVA (three times for 15 min each) and oocytes were immersed in PBS-PVA containing 2.5% (*v*/*v*) Tween 20 (GE Healthcare Co., Chicago, IL, USA) for 2 min at 24 °C. Blastocysts were washed with PBS-PVA (three times for 15 min each) again, incubated for 2 h in PBS-PVA containing 1% (*w*/*v*) BSA (PBS-BSA) at 4 °C and thereafter in blocking buffer (PBS-BSA containing 10% (*v*/*v*) goat serum) for 40 min at 24 °C. The blastocysts were incubated in primary antibody overnight at 4 °C. The primary antibodies used were an anti-CDX2 rabbit monoclonal antibody (1:500; BioGenex, Fremont, CA, USA) to detect the TE cells and an anti-NANOG mouse polyclonal antibody (1:500; Abcam, Cambridge, UK) to detect the ICM cells. Alexa Fluor 568-labeled goat anti-rabbit IgG and Alexa Fluor 488-labeled goat anti-mouse IgG (both 1:500; Thermo Fisher Scientific, Waltham, MA, USA) were used as secondary antibodies to detect the ICM and TE cells, respectively. DNA was stained with DAPI (2 μg/mL; Molecular Probes, Eugene, OR, USA).

### 2.10. Statistical Analysis

Statistical analysis was performed using the Statcel 3 software (OMS Ltd., Saitama, Japan). All experiments were analyzed using one-way analysis of variance (ANOVA). The Tukey–Kramer procedure was for multiple comparisons. The parameters calculated as percentages were subjected to arcsine transformation before performing ANOVA, and a *p* value of <0.05 was considered statistically significant.

## 3. Results

### 3.1. The Phenotype of Testes and Sperm Derived from Plcz1^−/−^ or Plcz1^−/−^eCS^−/−^ Male Mice 

**Figure 1:**
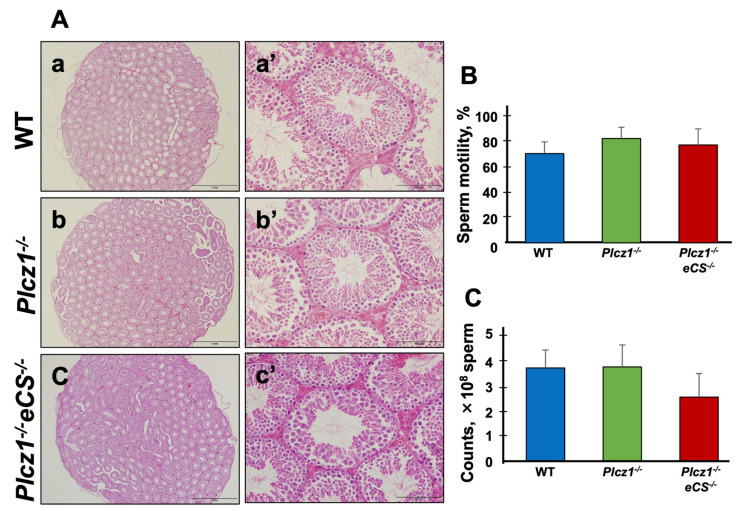
Phenotype of the *Plcz1^−^*^/*−*^ and *Plcz1^−^*^/*−*^*eCS^−^*^/*−*^ mice. (**A**) Histology of the *Plcz1^−^*^/*−*^ and *Plcz1^−^*^/*−*^*eCS^−^*^/*−*^ muse testes. Testes were collected from wildtype (WT) (**a**,**a’**), *Plcz1^−^*^/*−*^ (**b**,**b’**), and *Plcz1^−^*^/*−*^*eCS^−^*^/*−*^ mice (**c**,**c’**). Paraffin sections (6 µm) were stained with H&E. Scale bars: 1 mm (**a**–**c**) and 100 µm (**a’**–**c’**). (**B**) Motility of *Plcz1^−^*^/*−*^ and *Plcz1^−^*^/*−*^*eCS^−^*^/*−*^ mouse spermatozoa. Sperm motility was analyzed by the SMAS. *p* > 0.05. (**C**) Concentration of *Plcz1^−^*^/*−*^ and *Plcz1^−^*^/*−*^*eCS^−^*^/*−*^ mouse spermatozoa. Sperm motility was analyzed by the SMAS. *p* > 0.05.

At the beginning of the present study, we have checked the fertility of *eCS*^−/−^ male mice because a previous study reported that *eCS^−/−^* males were fertile, but their litter size was remarkably reduced in 6-month-old males [28]. However, under our experimental condition, we did not confirm a decrease in fertility in *eCS^−/−^* male mice even older than 30 weeks (7 months) of age as described above. Our results showed that the averages of pups were 7.9 (*n* = 21, 12–24 weeks from WT male), 7.2 (*n* = 20, 12–24 weeks from *eCS^−/−^* male) and 7.4 (*n* = 26, 32–50 weeks from *eCS^−/−^* male) (Supplementary Figure S1). Therefore, we used 12–24-week-old *Plcz1^−/−^eCS^−/−^* mice for the present study. The testes from *Plcz1^−^*^/*−*^ or *Plcz1^−^*^/*−*^*eCS^−^*^/*−*^ male mice were sectioned and stained with hematoxylin and eosin (H&E). Typical results are presented as Figure 1. In both the *Plcz1^−^*^/*−*^ and *Plcz1^−^*^/*−*^*eCS^−^*^/*−*^ testes, male germ cells and spermatozoa were confirmed (Figure 1b,c as they were in the wildtype [WT] testes (Figure 1a)). Indeed, the *Plcz1^−^*^/*−*^ and *Plcz1^−^*^/*−*^*eCS^−^*^/*−*^ male mice were not infertile. These results suggest that the genetic deletion of *Plcz1* and that of both *Plcz1* and *eCS* did not affect the production of sperm.

We next evaluated motility and concentration of sperm derived from *Plcz1^−^*^/*−*^ or *Plcz1^−^*^/*−*^*eCS^−^*^/*−*^ male mice. These results were shown in Figure 1B,C. The sperm motilities were 70.9 ± 9.2% (WT), 82.8 ± 9.0% (*Plcz1^−/−^*) and 77.7 ± 12.8% (*Plcz1^−/−^eCS^−/−^*). There were no significant differences among the groups (*p* > 0.05). The sperm concentrations were 3.7 ± 0.7 × 10^8^ sperm/mL (WT), 3.7 ± 0.9 × 10^8^ sperm/mL (*Plcz1^−/−^*) and 2.5 ± 0.9 × 10^8^ sperm/mL (*Plcz1^−/−^eCS^−/−^*). There were no significant differences among the groups (*p* > 0.05).

### 3.2. The Effects of the Different Regimens on the Activation and Embryonic Development of Oocytes Injected with Plcz1^−/−^ or Plcz1^−/−^eCS^−/−^ Sperm

**Figure 2:**
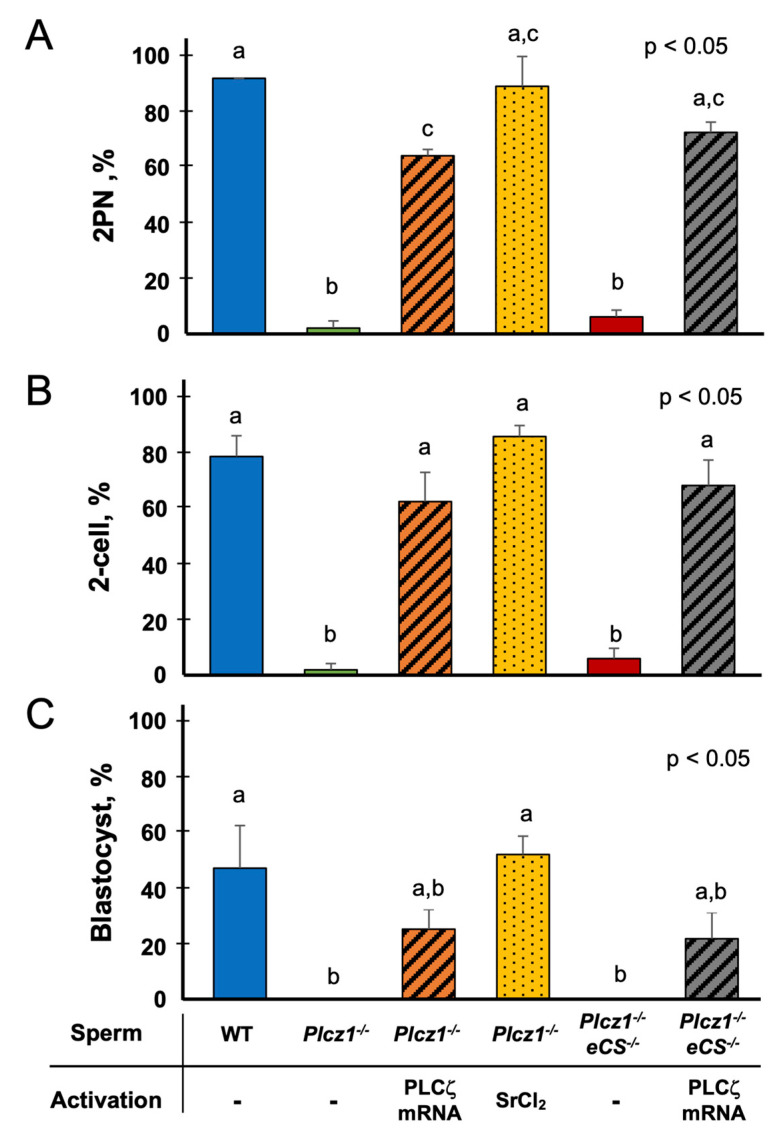
The in vitro developmental ability of embryos derived from *Plcz1^−^*^/*−*^ and *Plcz1^−^*^/*−*^*eCS^−^*^/*−*^ sperm with different activation protocols. The rates of 2 pronuclear (2PN)-stage (**A**), two-cell embryos (**B**), and blastocysts (**C**) from the embryos derived from *Plcz1^−^*^/*−*^ and *Plcz1^−^*^/*−*^*eCS^−^*^/*−*^ sperm with different activation protocols. Different superscript letters: *p* < 0.05.

As shown in Figure 2A, the injection of WT sperm into the MII oocytes resulted in the induction of pronuclear formation in most of the oocytes (92.1 ± 2.6%). However, only a few (2.0 ± 2.3% or 6.1 ± 3.7%) oocytes formed pronuclei when sperm derived from *Plcz1^−^*^/*−*^ or *Plcz1^−^*^/*−*^*eCS^−^*^/*−*^ mice were injected. A subsequent injection of PLCζ mRNA significantly improved the rates of pronuclear formation in oocytes derived from *Plcz1^−^*^/*−*^ (64.2 ± 10.8%) and *Plcz1^−^*^/*−*^*eCS^−^*^/*−*^ sperm (72.6 ± 5.4%). The additional treatment with SrCl_2_ also induced pronuclear formation in oocytes injected with *Plcz1^−^*^/*−*^ sperm (89.2 ± 2.4%).

The embryonic development of these oocytes is presented in Figure 2B,C. In the WT sperm-injected group, the percentages of two-cell embryos and blastocysts were 78.9 ± 7.5% and 47.4 ± 15.4%, respectively, and in the *Plcz1^−^*^/*−*^ sperm + PLCζ mRNA-injected group, the respective percentages were 62.7 ± 10.5% and 25.4 ± 7.1%. In the *Plcz1^−^*^/*−*^*eCS^−^*^/*−*^ sperm + PLCζ mRNA-injected group, the percentages of two-cell embryos and blastocysts were 68.5 ± 9.2% and 21.9 ± 9.5%, and those in the *Plcz1^−^*^/*−*^ sperm-injected + SrCl_2_-treated group were 86.2 ± 4.0% and 52.3 ± 6.6%, respectively. There were no significant differences in the percentages of two-cell embryos or blastocysts among these groups. On the other hand, very few oocytes developed to the two-cell stage in the *Plcz1^−^*^/*−*^ sperm-injected group (2.0 ± 2.3%) or in the *Plcz1^−^*^/*−*^*eCS^−^*^/*−*^ sperm-injected group (6.1 ± 3.7%), and none of the two-cell embryos reached the blastocyst stage in these two groups.

**Figure 3:**
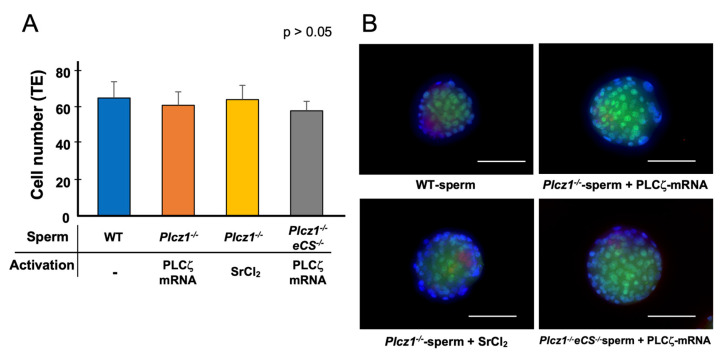
The cell number of TE at the blastocysts derived from *Plcz1^−^*^/*−*^ and *Plcz1^−^*^/*−*^*eCS^−^*^/*−*^ sperm with different activation protocols. (**A**) The cell numbers of TEs in the blastocysts derived from *Plcz1^−^*^/*−*^ and *Plcz1^−^*^/*−*^*eCS^−^*^/*−*^ sperm with different activation protocols. (**B**) Representative images of the embryos stained with CDX1 (Green) and NANOG (Red) antibodies. Scale bars: 100 µm.

The numbers of inner cell mass (ICM) and trophectoderms (TEs) in the blastocysts derived from *Plcz1^−^*^/*−*^ sperm or *Plcz1^−^*^/*−*^*eCS^−^*^/*−*^ sperm with different activation regimens are shown in Figure 3A,B. CDX2 and NANOG antibodies were used as markers for TE and ICM, respectively. The number of CDX2-positive cells were 66.4 ± 9.2 (WT), 62.3 ± 7.6 (*Plcz1^−^*^/*−*^ sperm + PLCζ mRNA-injected group), 65.5 ± 8.0 (*Plcz1^−^*^/*−*^*eCS^−^*^/*−*^ sperm + PLCζ mRNA-injected group), and 59.2 ± 5.3 (*Plcz1^−^*^/*−*^ sperm-injected + SrCl_2_-treated group). There were no significant differences in the number of CDX2-positive cells among the groups (*p* > 0.05). The numbers of NANOG-positive cells were 4.8 ± 0.8 (WT), 4.5 ± 1.7 (*Plcz1^−^*^/*−*^ sperm + PLCζ mRNA-injected group), 4.0 ± 1.0 (*Plcz1^−^*^/*−*^*eCS^−^*^/*−*^ sperm + PLCζ mRNA-injected group), and 4.4 ± 1.1 (*Plcz1^−^*^/*−*^ sperm-injected + SrCl_2_-treated group), with no significant differences among the groups.

The weights of the pups were 1.18 ± 0.04 g (WT), 1.34 ± 0.04 g (*Plcz1^−^*^/*−*^ sperm + PLCζ mRNA-injected group), 1.15 ± 0.09 g (*Plcz1^−^*^/*−*^ sperm-injected + SrCl_2_-treated group), and 1.09 ± 0.03 (*Plcz^−^*^/*−*^*eCS^−^*^/*−*^ sperm + PLCζ mRNA-injected group), with no significant differences among the groups. The weights of placenta were 0.12 ± 0.01 g (WT), 0.11 ± 0.02 g (*Plcz1^−^*^/*−*^ sperm + PLCζ mRNA-injected group), 0.09 ± 0.01 g (*Plcz1^−^*^/*−*^ sperm-injected + SrCl_2_-treated group), and 0.11 ± 0.01 g (*Plcz1^−^*^/*−*^*eCS^−^*^/*−*^ sperm + PLCζ mRNA-injected group, with no significant differences among the groups.

### 3.3. In Vivo Development of Embryos Derived from Plcz1^−/−^ or Plcz1^−/−^eCS^−/−^ Sperm by Different Activation Regimens

**Figure 4:**
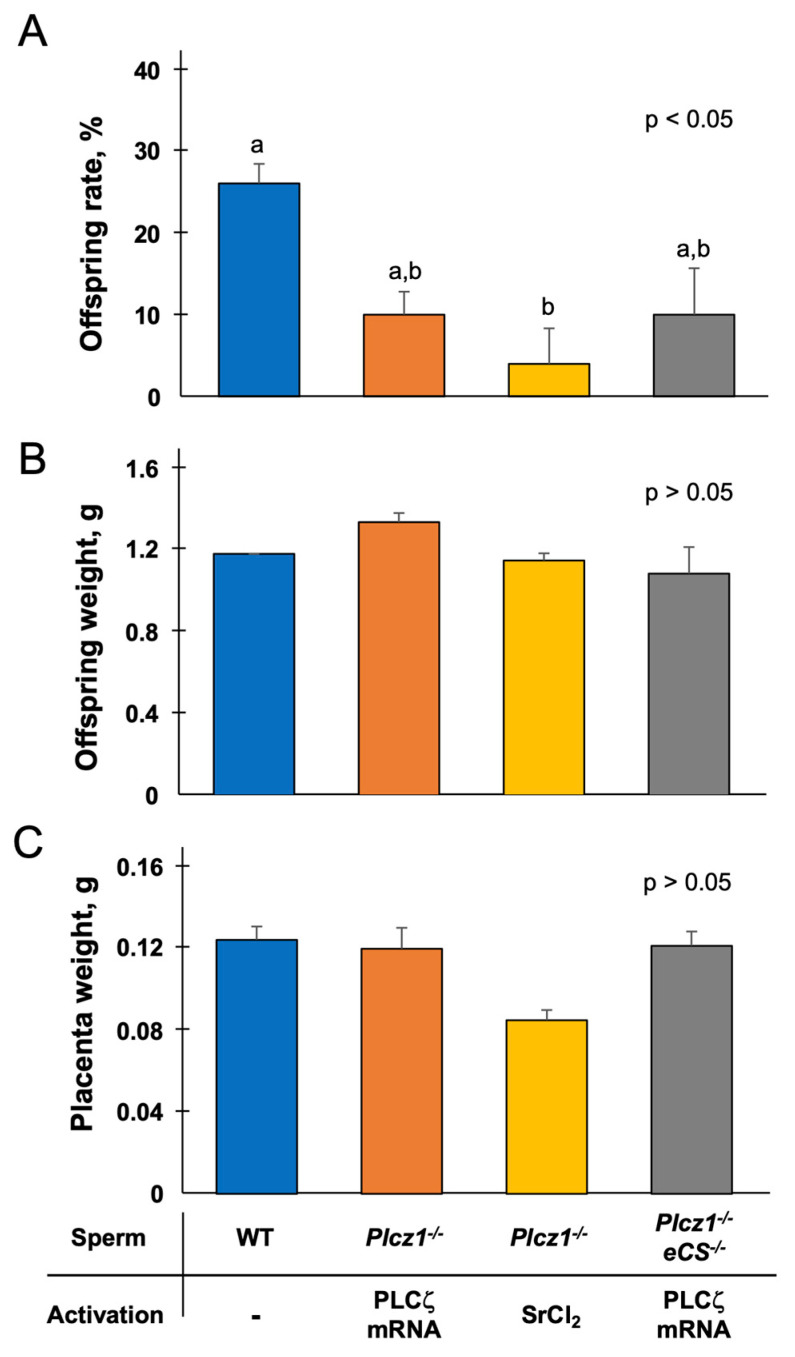
In vivo developmental ability of embryos derived from *Plcz1^−^*^/*−*^ and *Plcz1^−^*^/*−*^*eCS^−^*^/*−*^ sperm with different activation protocols. The offspring rates (**A**), body weights (**B**), and placenta weights (**C**) from the embryos derived from *Plcz1^−^*^/*−*^ and *Plcz1^−^*^/*−*^*eCS^−^*^/*−*^ sperm with different activation protocols after embryo transfer. Different superscript letters: *p* < 0.05.

**Figure 5:**
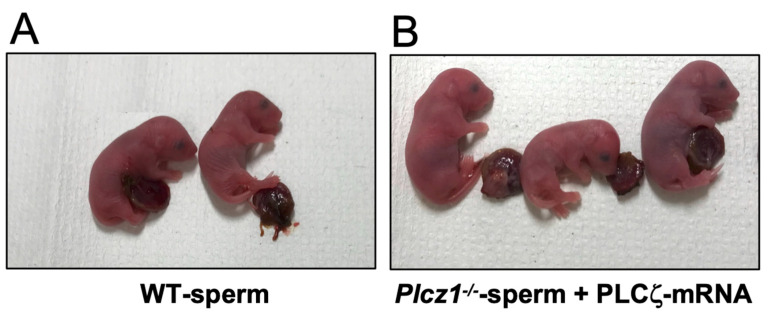
Pups derived from *Plcz1^−^*^/*−*^ and *Plcz1^−^*^/*−*^*eCS^−^*^/*−*^ sperm with different activation protocols. The representative images of offspring derived from WT sperm (**A**) and *Plcz1^−^*^/*−*^ sperm + PLCζ mRNA-injected groups (**B**).

To examine the ability to develop to term, we transferred two-cell embryos to the oviducts of the recipients. The results are shown in Figure 4A–C. The rates of offspring were 26.0 ± 2.4% (23 pups/90 embryos) in the WT group, 10.0 ± 2.8% (9 pups/91 embryos) in the *Plcz1^−^*^/*−*^ sperm + PLCζ mRNA-injected group, 4.0 ± 4.3% (2 pups/50 embryos) in the *Plcz1^−^*^/*−*^ sperm-injected + SrCl_2_-treated group, and 10.0 ± 5.7% (6 pups/61 embryos) in the *Plcz1^−^*^/*−*^*eCS^−^*^/*−*^ sperm + PLCζ mRNA-injected group. All pups looked healthy and morphological abnormality was not confirmed (Figure 5). The rate of offspring in the *Plcz1^−^*^/*−*^ sperm-injected + SrCl_2_-treated group was significantly lower than that in the WT group (*p* < 0.05).

## 4. Discussion

PLCζ has been suspected to be the sperm factor that induces oocyte activation [13,33], at least in mammals. Nozawa et al. [20] demonstrated that *Plcz1^−^*^/*−*^ mouse spermatozoa failed to induce Ca^2+^ changes in ICSI; however, healthy pups were obtained after transplantation of the *Plcz1^−^*^/*−*^ sperm-derived embryos when PLCζ mRNA was injected into the embryos. Our present results also demonstrated the successful production of embryos derived from *Plcz1^−^*^/*−*^ spermatozoa with the injection of PLCζ mRNA, similar to the findings described by Nozawa et al. These results strongly suggest that (i) PLCζ works per se as a sperm factor, and (ii) its function fully supports the ability to develop to term.

Extra-mitochondrial citrate synthase (eCS), which has been described as another sperm factor [28], was first reported as a sperm factor in newts; the microinjection of eCS into unfertilized newt eggs induced a transient increase in intracellular calcium [34]. A later study using mice showed that (i) eCS was expressed in the sperm head even after the acrosome reaction, and (ii) the injection of eCS mRNA into MII oocytes induced calcium oscillations [28]. These results suggested that eCS is suitable as the sperm factor (as is PLCζ). Our present results demonstrated that an injection of PLCζ mRNA also rescued the developmental ability of the embryos derived from *Plcz1^−^*^/*−*^*eCS^−^*^/*−*^ sperm in addition to *Plcz1^−^*^/*−*^ sperm. In an earlier investigation, the injection of *eCS^−^*^/*−*^ spermatozoa delayed the calcium oscillations, but *eCS^−^*^/*−*^ male mice were fertile [28]. Although only old *eCS^−^*^/*−*^ male mice (more than 24 weeks) showed the decrease in fertility [28], the results from our study demonstrated that the decreased fertility of *eCS^−^*^/*−*^ male mice (even 32–50 weeks) was not observed (Supplementary Figure S1). We do not have any explanation about the difference, but in this study, we used 12–24-week-old male mice as the *Plcz1^−^*^/*−*^*eCS^−^*^/*−*^ group. In addition, our preliminary study showed that even *Plcz1^−^*^/*−*^*eCS^−^*^/*−*^ male mice were also not infertile and spermatozoa were confirmed in the testes from *Plcz1^−^*^/*−*^*eCS^−^*^/*−*^ male mice. Sperm motility and concentration in *Plcz1^−^*^/*−*^ and *Plcz1^−^*^/*−*^*eCS^−^*^/*−*^ groups were also similar to the WT group. In this study, we have not checked the fertility of embryos derived from *Plcz1^−/−^eCS^−/−^* sperm with old age and cannot rule out the possibility that sperm derived from *Plcz1^−/−^eCS^−/−^*-aged males can have altered fertilization ability in comparison to the young ones. Further experiments will be conducted to clarify the possibility. These results indicate that eCS is not essential for development to term, at least in the young mouse.

On the other hand, oocytes injected with *Plcz1^−^*^/*−*^ or *Plcz1^−^*^/*−*^*eCS^−^*^/*−*^ sperm did not form pronuclei if they were not treated with additional treatment. Although the intracellular calcium concentration was not measured in this experiment, previous studies already reported that when sperm derived from *Plcz1^−^*^/*−*^ male mice were used for ICSI, there was no increase in intracellular calcium in the oocytes [19,20]. Based on these facts, it is considered in the present study that the rises of intracellular calcium (including calcium oscillations) was completely suppressed in the oocytes injected with *Plcz1^−^*^/*−*^ or *Plcz1^−^*^/*−*^*eCS^−^*^/*−*^ sperm, resulting in the failure of pronuclear formation. As shown in the previous studies and our unpublished observation, since PLCζ-independent and membrane-fusion-dependent raises of intracellular calcium mechanisms exist, *Plcz1^−^*^/*−*^ or *Plcz1^−^*^/*−*^*eCS^−^*^/*−*^ can be fertilized and induce pronuclear formation in IVF.

The treatment with SrCl_2_ in the present study was also demonstrated to rescue the developmental ability of *Plcz1^−^*^/*−*^-spermatozoa-derived embryos. Although the rescue experiment using SrCl_2_ was not performed for *Plcz1^−^*^/*−*^*eCS^−^*^/*−*^-spermatozoa-derived embryos, the results would be similar to that from *Plcz1^−^*^/*−*^-spermatozoa-derived embryos. Treatment with SrCl_2_ is widely used to induce oocyte activation for SCNT and ROSI in several mammalian species [35,36,37,38]. Although we observed that the developmental ability of the *Plcz1^−^*^/*−*^-sperm-derived embryos treated with SrCl_2_ was greater than that of the embryos injected with PLCζ mRNA, the cell numbers of the blastocyst were significantly decreased in the *Plcz1^−^*^/*−*^ + SrCl_2_ group compared to that in the *Plcz1^−^*^/*−*^ + PLCζ mRNA group. In addition, the offspring rates when PLCζ mRNA was used were superior to those achieved with SrCl_2_ for the activation of *Plcz1^−^*^/*−*^ sperm-derived oocytes. In the previous study [39], using fertilized oocytes that have undergone the first few calcium oscillations, they developed procedures that result either in inhibiting or stimulating the natural pattern of calcium signaling of inseminated oocytes. Although the incidence of development to the blastocyst stage is unaltered by these procedures, fewer offspring are born following embryo transfer, indicating that developmental competence of the blastocysts is reduced [39]. It has been shown that treatment with SrCl_2_ can induce the calcium-oscillations-like phenomenon that generally occurs during fertilization in mammals [27,40]. It is still unclear whether there is a difference between PLCζ-induced oscillations and SrCl_2_-induced oscillations-like phenomenon. At least, PLC is a hydrolytic enzyme that produces IP_3_ and DG but not SrCl_2_. Although further experiments will be required, it seems to be a possibility that the calcium oscillation-like phenomenon caused by SrCl_2_ does not fully reproduce PLCζ-derived calcium oscillations. This difference may result in the low rate of the pups derived from *Plcz1^−^*^/*−*^ sperm-injected + SrCl_2_-treated group. In light of these results, we speculate that PLCζ has essential roles in not only oocyte activation but also embryonic development to term. Further investigations will clarify the detailed functions of PLCζ during fertilization in mammals.

## 5. Conclusions

Our present findings demonstrate that additional treatment (i.e., an injection of PLCζ mRNA or SrCl_2_ treatment) inducing a calcium-oscillation-like phenomenon can rescue the developmental ability of *Plcz1^−^*^/*−*^-spermatozoa-derived embryos. Many reproductive technologies such as ICSI and round spermatid injection (ROSI) have been developed and used in various research and clinical areas to date. The successful production of cloned animals derived from somatic cell nuclear transfer (SCNT) was also reported in several mammalian species [21,22,23,24].

At least in mice, treatment with strontium chloride (SrCl_2_) is applied for inducing oocyte activation in ROSI [25] and SCNT [26] because it is believed that SrCl_2_ treatment can produce fertilization events such as calcium oscillations in oocytes [27]. Our present results suggest injection of PLCζ mRNA seems to be more suitable for oocyte activation compared with SrCl_2_. However, it is required that the subsequent normality of the offspring derived from additional activation treatments will be evaluated in further investigations.

In addition, it has been indicated that male infertility in humans depends on the genetic mutation of *Plcz1^−^*^/*−*^, which causes a loss of calcium oscillations, resulting in failed pregnancy after ICSI [41,42,43]. Since injecting PLCζ mRNA will raise ethical issues in a human clinical field, it will be necessary to consider the development of artificial activation methods that more closely resemble normal fertilization in the future. Our current observations will contribute to the improvement of PLCζ-dependent male infertility.

## Data Availability

The datasets analyzed during the current study are available from the corresponding author upon reasonable request.

## Supplementary Materials

The following supporting information can be downloaded at: https://www.mdpi.com/article/10.3390/life13040980/s1. Figure S1. Fertility of young and aged *eCS^−/−^* mice.

## References

[1] Ito J., Parrington J., Fissore R.A. (2011). PLCζ and its role as a trigger of development in verte-brates. Mol. Reprod. Dev..

[2] Ducibella T., Fissore R. (2008). The roles of Ca^2+^, downstream protein kinases, and oscillatory signaling in regulating fertilization and the activation of development. Dev. Biol..

[3] Schultz R.M., Kopf G.S. (1995). Molecular basis of mammalian egg activation. Curr. Top. Dev. Biol..

[4] Saunders C.M., Larman M.G., Parrington J., Cox L.J., Royse J., Blayney L.M., Swann K., Lai F.A. (2002). PLC zeta: A sperm-specific trigger of Ca(2+) oscillations in eggs and embryo development. Development.

[5] Ito M., Shikano T., Oda S., Horiguchi T., Tanimoto S., Awaji T., Mitani H., Miyazaki S. (2008). Difference in Ca^2+^ oscillation-inducing activity and nuclear translocation ability of PLCZ1, an egg-activating sperm factor candidate, between mouse, rat, human, and medaka fish. Biol. Reprod..

[6] Ito M., Shikano T., Kuroda K., Miyazaki S. (2008). Relationship between nuclear sequestration of PLCzeta and termination of PLCzeta-induced Ca^2+^ oscillations in mouse eggs. Cell Calcium.

[7] Cox L.J., Larman M.G., Saunders C.M., Hashimoto K., Swann K., Lai F.A. (2002). Sperm phospholipase Czeta from humans and cynomolgus monkeys triggers Ca^2+^ oscillations, activation and development of mouse oocytes. Reproduction.

[8] Cooney M.A., Malcuit C., Cheon B., Holland M.K., Fissore R.A., D’Cruz N.T. (2010). Specific differences in the activity and nuclear localization of murine and bovine phospholipase C zeta 1. Biol. Reprod..

[9] Malcuit C., Knott J.G., He C., Wainwright T., Parys J.B., Robl J.M., Fissore R.A. (2005). Fertilization and inositol 1,4,5-trisphosphate (IP3)-induced calcium release in type-1 inositol 1,4,5-trisphosphate receptor down-regulated bovine eggs. Biol. Reprod..

[10] Kurokawa M., Sato K., Fissore R.A. (2004). Mammalian fertilization: From sperm factor to phospholipase Czeta. Biol. Cell.

[11] Yoneda A., Kashima M., Yoshida S., Terada K., Nakagawa S., Sakamoto A., Hayakawa K., Suzuki K., Ueda J., Watanabe T. (2006). Molecular cloning, testicular postnatal expression, and oocyte-activating potential of porcine phospholipase Czeta. Reproduction.

[12] Sato K., Wakai T., Seita Y., Takizawa A., Fissore R.A., Ito J., Kashiwazaki N. (2013). Molecular characteristics of horse phospholipase C zeta (PLCζ). Anim. Sci. J..

[13] Nakai M., Ito J., Suyama A., Kageyama A., Tobari Y., Kashiwazaki N. (2020). Phospholipase Czeta (PLCzeta) versus postacrosomal sheath WW domain-binding protein (PAWP): Which molecule will survive as a sperm factor?. Anim. Sci. J..

[14] Kouchi Z., Fukami K., Shikano T., Oda S., Nakamura Y., Takenawa T., Miyazaki S. (2004). Recombinant phospholipase Czeta has high Ca^2+^ sensitivity and induces Ca^2+^ oscillations in mouse eggs. J. Biol. Chem..

[15] Heytens E., Parrington J., Coward K., Young C., Lambrecht S., Yoon S., Fissore R.A., Hamer R., Deane C.M., Ruas M. (2009). Reduced amounts and abnormal forms of phospholipase C zeta (PLCzeta) in spermatozoa from infertile men. Hum. Reprod..

[16] Yoon S., Jellerette T., Salicioni A.M., Lee H.C., Yoo M., Coward K., Parrington J., Grow D., Cibelli J.B., Visconti P.E. (2008). Human sperm devoid of PLC, zeta 1 fail to induce Ca(2+) release and are unable to initiate the first step of embryo development. J. Clin. Invest..

[17] Kashir J., Konstantinidis M., Jones C., Heindryckx B., De Sutter P., Parrington J., Wells D., Coward K. (2012). Characterization of two heterozygous mutations of the oocyte activation factor phospholipase C zeta (PLCzeta) from an infertile man by use of minisequencing of individual sperm and expression in somatic cells. Fertil. Steril..

[18] Escoffier J., Lee H.C., Yassine S., Zouari R., Martinez G., Karaouzene T., Coutton C., Kherraf Z., Halouani L., Triki C. (2016). Homozygous mutation of PLCZ1 leads to defective human oocyte activation and infertility that is not rescued by the WW-binding protein PAWP. Hum. Mol. Genet..

[19] Hachem A., Godwin J., Ruas M., Lee H.C., Ferrer Buitrago M., Ardestani G., Bassett A., Fox S., Navarrete F., de Sutter P. (2017). PLCζ is the physiological trigger of the Ca^2+^ oscillations that induce embryogenesis in mammals but conception can occur in its absence. Development.

[20] Nozawa K., Satouh Y., Fujimoto T., Oji A., Ikawa M. (2018). Sperm-borne phospholipase C zeta-1 ensures monospermic fertilization in mice. Sci. Rep..

[21] Wilmut I., Schnieke A.E., McWhir J., Kind A.J., Campbell K.H.S. (2007). Viable offspring derived from fetal and adult mammalian cells. Cloning Stem. Cells.

[22] Kato Y., Tani T., Sotomaru Y., Kurokawa K., Kato J., Doguchi H., Yasue H., Tsunoda Y. (1998). Eight calves cloned from somatic cells of a single adult. Science.

[23] Wakayama T., Perry A.C., Zuccotti M., Johnson K.R., Yanagimachi R. (1998). Full-term development of mice from enucleated oocytes injected with cumulus cell nuclei. Nature.

[24] Onishi A., Iwamoto M., Akita T., Mikawa S., Takeda K., Awata T., Hanada H., Perry A.C. (2000). Pig cloning by microinjection of fetal fibroblast nuclei. Science.

[25] Kishigami S., Wakayama S., Nguyen V.T., Wakayama T. (2004). Similar time restriction for intracytoplasmic sperm injection and round spermatid injection into activated oocytes for efficient offspring production. Biol. Reprod..

[26] Ogura A. (2017). Cloning Mice. Cold Spring Harb. Protoc..

[27] Kishigami S., Wakayama T. (2007). Efficient strontium-induced activation of mouse oocytes in standard culture media by chelating calcium. J. Reprod. Dev..

[28] Kang W., Harada Y., Yamatoya K., Kawano N., Kanai S., Miyamoto Y., Nakamura A., Miyado M., Hayashi Y., Kuroki Y. (2020). Extra-mitochondrial citrate synthase initiates calcium oscillation and suppresses age-dependent sperm dysfunction. Lab. Invest..

[29] Yamamoto Y., Hirose N., Kamimura S., Wakayama S., Ito J., Ooga M., Wakayama T. (2020). Production of mouse offspring from inactivated spermatozoa using horse PLCζ mRNA. J. Reprod. Dev..

[30] Kimura Y., Yanagimachi R. (1995). Intracytoplasmic sperm injection in the mouse. Biol. Reprod..

[31] Chatot C.L., Ziomek C.A., Bavister B.D., Lewis J.L., Torres I. (1989). An improved culture medium supports development of random-bred 1-cell mouse embryos in vitro. J. Reprod. Fertil..

[32] Hirose N., Wakayama S., Inoue R., Ito J., Ooga M., Wakayama T. (2020). Birth of offspring from spermatid or somatic cell by co-injection of PLCζ-cRNA. Reproduction.

[33] Swann K. (2022). Sperm Factors and Egg Activation: PLCzeta as the sperm factor that activates eggs: 20 years on. Reproduction.

[34] Harada Y., Matsumoto T., Hirahara S., Nakashima A., Ueno S., Oda S., Miyazaki S., Iwao Y. (2007). Characterization of a sperm factor for egg activation at fertilization of the newt Cynops pyrrhogaster. Dev. Biol..

[35] Chesné P., Adenot P.G., Viglietta C., Baratte M., Boulanger L., Renard J. (2002). Cloned rabbits produced by nuclear transfer from adult somatic cells. Nat. Biotechnol..

[36] Zhou Q., Renard J., Le Friec G., Brochard V., Beaujean N., Cherifi Y., Fraichard A., Cozzi J. (2003). Generation of fertile cloned rats by regulating oocyte activation. Science.

[37] Ogonuki N., Tsuchiya H., Hirose Y., Okada H., Ogura A., Sankai T. (2003). Pregnancy by the tubal transfer of embryos developed after injection of round spermatids into oocyte cytoplasm of the cynomolgus monkey (Macaca fascicularis). Hum. Reprod..

[38] Hirabayashi M., Kato M., Kitada K., Ohnami N., Hirao M., Hochi S. (2009). Activation regimens for full-term development of rabbit oocytes injected with round spermatids. Mol. Reprod. Dev..

[39] Ozil J., Banrezes B., Tóth S., Pan H., Schultz R.M. (2006). Ca^2+^ oscillatory pattern in fertilized mouse eggs affects gene expression and development to term. Dev. Biol..

[40] Wakai T., Zhang N., Vangheluwe P., Fissore R.A. (2013). Regulation of endoplasmic reticulum Ca(2+) oscillations in mammalian eggs. J. Cell Sci..

[41] Anifandis G., Michopoulos A., Daponte A., Chatzimeletiou K., Simopoulou M., Messini C.I., Polyzos N.P., Vassiou K., Dafopoulos K., Goulis D.G. (2019). Artificial oocyte activation: Physiological, pathophysiological and ethical aspects. Syst. Biol. Reprod. Med..

[42] Saleh A., Kashir J., Thanassoulas A., Safieh-Garabedian B., Lai F.A., Nomikos M. (2020). Essential Role of Sperm-Specific PLC-Zeta in Egg Activation and Male Factor Infertility: An Update. Front. Cell Dev. Biol..

[43] Thanassoulas A., Swann K., Lai F.A., Nomikos M. (2022). Sperm Factors and Egg Activation: The structure and function relationship of sperm PLCZ1. Reproduction.

