# Supplementary material for: Successful Production of Offspring Derived from Phospholipase C Zeta-Deficient Sperm by Additional Artificial Activation
Source: Life (Basel). 2023 Apr 10;13(4):980. doi: 10.3390/life13040980 (PMC10143324; doi:10.3390/life13040980)
Supplement: Supplementary file 1 [file life-13-00980-s001.zip › life-2255579-supplementary.pdf]

Supplymental Figure S1

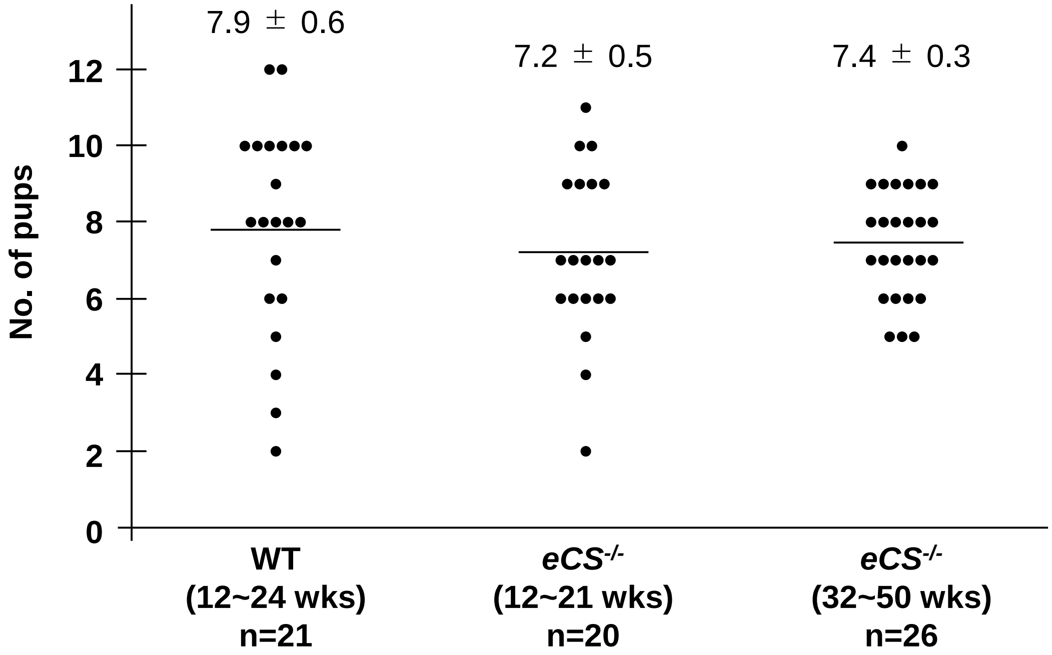

Figure S1. Fertility of young and aged *eCS*$^{-/-}$ mice. WT group was mated wild-type female and male mice. *eCS*$^{-/-}$ group was mated wild-type female mice with young or old *eCS*$^{-/-}$ male mice. Copulation was confirmed by checking for vaginal plugs every morning (9:00-11:00) and the number of pups were counted after parturition (20-21 days after plug confirmation). Average number of offspring was showed by means ± S.E.M. There were no significant differences ($P > 0.05$).
